# The *Candida quercitrusa* strain Cq-C08 induces plant resistance to root-knot nematodes

**DOI:** 10.3389/fmicb.2025.1546583

**Published:** 2025-04-17

**Authors:** Cuihua Lu, Erfeng Li, Rui Liu, Nv Chang, Yuqing Lai, Yue Wu, Weilong Wu, Zhukan Chen, Jian Ling, Jianlong Zhao, Zhenchuan Mao

**Affiliations:** ^1^College of Horticulture and Landscape Architecture, Tianjin Agricultural University, Tianjin, China; ^2^State Key Laboratory of Vegetable Biobreeding, Institute of Vegetables and Flowers, Chinese Academy of Agricultural Sciences, Beijing, China; ^3^Hangzhou Fuyang District Agriculture and Rural Bureau, Hangzhou, China

**Keywords:** root-knot nematodes, *Candida quercitrusa*, biocontrol, induced resistance, immune regulation

## Abstract

**Introduction:**

Root-knot nematodes (RKNs), belonging to the genus Meloidogyne, are plant parasitic nematodes with a broad host range, causing substantial economic losses annually. The selection and utilization of novel biological control resources are crucial for managing RKNs diseases.

**Methods:**

This study isolated *Candida quercitrusa* Cq-C08 from rhizosphere soil, which the efficacy of Cq-C08 against *Meloidogyne incognita* was investigated through laboratory experiments, pot and plot trials, and analysis of the transcriptomic data from cucumber roots treated with Cq-C08.

**Results and discussion:**

This study isolated *Candida quercitrusa* Cq-C08 from rhizosphere soil, and a series of experiments confirmed that the fermentation broth had a lethal rate of 100% against *M. incognita* J2s within 12 h and exhibited a significant repellent effect on the nematodes. In pot and plot tests, the strain Cq-C08 achieved a control effect over 50% against *M. incognita* and significantly promoted cucumber (*Cucumis sativus*, Zhongnong No. 6, China) growth. Inoculation experiments confirmed that the Cq-C08 strain could activate key immune signaling pathways of salicylic acid (SA) and jasmonic acid (JA). Split-root tests showed significant induced resistance of cucumber to *M. incognita* by 32.3%. Comparative transcriptome analysis confirmed that strain Cq-C08 could regulate the host’s basal immune response and oxidative burst response through SA, JA, and ethylene (ET) signaling pathways, and alter secondary metabolism, activating the synthesis of cucurbitacin and auxins, which promotes plant immune regulation and growth. These results prove that *C. quercitrusa* Cq-C08 has high control effects against *M. incognita* and the potential to be developed into a biological control product against root-knot nematodes.

## 1 Introduction

Root-knot nematodes (RKNs, *Meloidogyne* spp.) are soil-borne pathogens constitute a significant threat to a diverse range of agricultural crops, including important horticultural crops, solanaceous, and cucurbitaceous plants (Perry et al., 2009). The economic losses due to plant-parasitic nematodes damage to crops amount to $125 billion annually, of which RKNs is the most important species (Tapia-Vázquez et al., 2022). Common species include *M. incognita*, *M. hapla*, *M. javanica*, *M. arenaria* and *M. enterolobii*, with *M. incognita* being the most widely distributed and damaging (Jones et al., 2013). Second-stage juveniles (J2s) of RKNs are the infective stage, which migrate to host-plant roots. They form hypertrophied feeding cells called giant cells, providing nutrient source for the nematodes and significantly weakening the plant’s ability to absorb water and nutrients, causing an imbalance in the plant’s normal physiological and metabolic functions (Eves-van Den Akker, 2021; Favery et al., 2016; Siddique et al., 2022). Additionally, the wounds left by nematode invasion provide an entry point for other pathogenic microorganisms, increasing the risk of plants being infected with multiple diseases. This leads to stunted plant growth, yellowing, reduced fruit yield, and severely affects agricultural production (Williamson, 1998).

In most cases, chemical nematicides have long dominated the arsenal against root-knot nematodes (RKNs) (Phani et al., 2021). Although these synthetic substances prove effective initially, they are severely constrained by multiple issues. These include their persistence in the environment, toxicity to non-target organisms, and the development of resistance within nematode populations (Collange et al., 2011). Such drawbacks have urgently necessitated a paradigm shift toward biological control strategies. These strategies capitalize on the natural antagonistic traits of microorganisms to manage RKNs in a more sustainable and eco-friendly fashion (Lartey et al., 2023). Biological control has emerged as one of the safest and alternative effective measures for pathogen prevention and control. By harnessing the microorganisms present in the soil, it can effectively kill or restrict pathogens, thus circumventing problems like toxic residues and environmental pollution that are often associated with chemical control methods (Syed Ab Rahman et al., 2018). A diverse range of biocontrol agents isolated for RKN control including various microbial groups, such as fungi, bacteria, and yeasts (Khan et al., 2023; Liu et al., 2019). Studies have revealed that plant growth-promoting bacteria and antagonistic fungi not only directly combat specific pathogens but also possess the capacity to induce plant resistance, thereby enhancing the plants’ resilience against pests and nematodes (Liu et al., 2023; Yin et al., 2021; Zhuang et al., 2021). Induced resistance (IR) in plants refers to a state of enhanced defense capability that plants develop after perceiving specific stimuli, which can effectively resist the upcoming pathogens and pests. This induced resistance includes systemic acquired resistance (SAR) and localized acquired resistance (LAR) (De Kesel et al., 2021). Nevertheless, current research efforts are more extensively focused on bacteria and fungi, with examples including *Bacillus*, *Penicillium*, *Trichoderma*, and others (Li et al., 2015; Schouten, 2016; Sharma et al., 2022).

Yeast, as a single-celled eukaryotic organism, is characterized by its ease of cultivation, long survival time in soil, and strong stress resistance. There is evidence showing that yeasts can promote plant growth by solubilizing phosphates and also help plants resist pathogens (Ma et al., 2023; Mukherjee et al., 2020). In a recent study, yeasts have been used to prevent diseases in vegetables, fruits, and other crops, especially in the control of RKN diseases (Díaz et al., 2020). *Pseudozyma flocculosa*, a biocontrol agent against powdery mildew, secretes an effector Pf2826 crucial for its biocontrol activity. The effector was found to interact with barley pathogenesis-related proteins and a powdery mildew effector protein, suggesting a novel mode of action for a biocontrol agent involving effector-mediated manipulation of host-pathogen interactions (Santhanam et al., 2023). *Candida quercitrusa* is a novel biocontrol strain with unique biological characteristics and metabolic products. It was report that the yeast strain *C. quercitrusa* Cq-1 had strong anti-oomycete activity on *Phytophthora infestans*, primarily through the production of the natural volatile organic compound 2-phenylethanol (Lu et al., 2023). Moreover, *C. quercitrusa* exhibits significant inhibitory effects on various pathogenic fungi, capable of disrupting their cell walls by secreting chitinase and 2-phenylethanol, thereby achieving antibacterial and disease prevention effects. Additionally, it can suppress the growth of pathogenic bacteria through mechanisms such as antagonism and induced resistance (Liao et al., 2025). It was report that the yeast strain *Papiliotrema terrestris* PT22AV not only significantly reduced nematode populations and gall formation, but also enhanced tomato growth and yield, suggesting its potential as a biocontrol agent in sustainable RKNs management (D’Addabbo et al., 2024). However, this work did not explore the specific molecular mechanism of PT22AV on RKNs, such as whether it inhibited nematodes parasitism by producing nematode toxins, inducing plant resistance, or competing for nutrients. Considering that yeasts are generally safe for the environment and non-target organisms, making them a sustainable alternative to chemical nematicides, more and high efficient yeasts strains need to be identified for controlling RKNs disease, and their functional mechanisms require further analysis for better application. Build upon this, we identified a yeast strain *C. quercitrusa* Cq-C08, which showed high nematicidal activity in lab experiments, in pot assays and also in field assays. We provide evidence that strain Cq-C08 could reduce nematode infectivity, induce resistance of plants and promote plant development through modulating plant immune responses and hormone synthesis. These findings lay a foundation for subsequent development of biocontrol agents based on Cq-C08 to control RKNs in agricultural production.

## 2 Materials and methods

### 2.1 Plant and nematode materials

Cucumber variety Zhongnong No. 6 was a cultivated variety produced by the Institute of Vegetables and Flowers, Chinese Academy of Agricultural Sciences (Beijing, China). For preparing hydroponic cucumber seedlings, seeds were surface sterilized with 0.5% NaOCl and placed in pots with filter paper and sterilized water. The pots were kept in the dark at 28°C for 3 days, followed by a 10-day period with a photoperiod of 16 h light and 8 h darkness.

*Meloidogyne incognita* was reproduced initially from a single egg mass on tomato plants (*Solanum lycopersicum* var. “Moneymaker”) (Zhao et al., 2024), and reproduced on pepper plants (*Capsicum annuum* var. “Qiemen”) in a greenhouse at 28°C. Egg masses of *M. incognita* were collected from the plant roots, hatched in water at 28°C, and the infective *M. incognita* J2s were collected after 24 h for subsequent analyses.

### 2.2 Isolation and identification of strain Cq-C08

Rhizosphere soil samples were collected from an orchard in Langfang City (Hebei, China; 116.70°E, 39.53°N). Yeast strains were isolated using the soil solution dilution method. Fifty grams of soil were added to 1 L distilled water, shaken for 3 min, and then 1 mL supernatant was taken and diluted 100 times with sterile water. A 50 μL of the diluted liquid was spread on a YPDA plate and incubated in an incubator at 28°C. Single colonies were selected for identification.

Morphological identification was performed on the strain according to the “Fungal Identification Manual.” A single colony was used as a template, and universal fungal primer NLF/R (Supplementary Table 1) were used to amplify the 26S rRNA sequence. The following conditions were used: denaturation at 95°C for 3 min, followed by 34 cycles at 95°C for 15 s, 55°C for 15 s, and 72°C for 15 s, and extension at 72°C for 5 min. Bidirectional sequencing was performed on the amplified product sequences. The sequence results were analyzed by using the NCBI BLAST tool.

### 2.3 Nematicidal activity of strain Cq-C08 *in vitro*

Twenty-four-well culture plates were used to evaluate the nematicidal activity of strain Cq-C08 culture solution. Strain Cq-C08 was inoculated in liquid YPDA medium and cultured at 28°C with a shaking speed of 180 rpm for 3 days. The fermentation broth (OD600 = 1.0) was used for testing. The supernatant of the fermentation broth was collected by centrifugation at 10,000 rpm for 3 min.

One milliliter of fermentation broth or supernatant was added to each well of a 24-well plate, and then 10 μL nematode suspension containing 100 J2s was added to each well. Water and YPDA were used as controls. Each treatment included 10 replicates, and the assays were repeated three times. The 24-well plates were incubated at 28°C for 12 h, and then nematode mortality rates were observed. Nematodes were considered dead if they did not move upon stimulation with 1 N NaOH (Chen and Dickson, 2000). J2s mortality was calculated and photographed to record the different body morphology of nematodes. Corrected mortality rate (%) = [(mortality rate of treatment - mortality rate of control)/(1 - mortality rate of control)] × 100.

### 2.4 Verify the control effect of strain Cq-C08 on *M. incognita*

In pot assays, cucumber seedlings at the one-leaf stage were transplanted into pots (10 cm × 10 cm) filled with culture medium (peat/vermiculite, 2:1) and grown in a greenhouse at 28°C. Inoculation treatment was carried out at the two-leaf stage. Each pot was inoculated with 15 mL strain Cq-C08 fermentation broth, 10% Fosthiazate granule, or water, respectively. After 24 h, 500 *M. incognita* J2s suspension (1 mL) was inoculated into each pot. Each treatment consisted of 10 pots, and the assays were repeated three times. Cucumber galls and egg masses were counted at 35 days after *M. incognita* inoculation. Control effect (%) = 1 - (number of galls on treated root/number of galls on control root) × 100%.

The plot assays were conducted in greenhouses of Langfang City (Hebei, China), with two plot trials completed in 2022 and 2023, respectively. Each plot was 6.6 m^2^, and 30 seedlings were planted in each plot. Before transplanting of cucumber seedlings, the population densities of *M. incognita* during two test periods in Langfang were detected using the shallow dish method (Hussey and Barker, 1973). Each plant was inoculated with 500 mL strain Cq-C08 fermentation broth (OD600 = 1.5) diluted 10 times with water. Additionally, 10% Fosthiazate granule was applied as a nematicide treatment at a rate of 30 kg/ha, and water mock was used as a control. Each treatment was repeated three times independently. The incidence of the disease was investigated and the control effect of strain Cq-C08 was calculated. Moreover, the height of plants was measured to analyze the effect of strain Cq-C08 on cucumber growth. The disease index was rated on a scale of 1-5, with 1 = no galls, 2 = 1–25% of roots with galls, 3 = 26–50% with galls, 4 = 51–75% with galls, and 5 = over 75% with galls. Disease index = [Σ(grade of galls × number of cucumbers at that grade)]/(total number of cucumbers × highest grade of galls) × 100; Control effect (%) = (disease index of control group - disease index of treated group)/(disease index of control group) × 100%.

### 2.5 *M. incognita* J2s attraction assays

The nematode attraction assays were conducted according to previous reported methods with some modifications (Cao et al., 2019; Tsai et al., 2021). Petri dishes (width = 9 cm) were divided into six equal, and covered with 0.5% water agar. Then, 20 μL of the strain Cq-C08 fermentation broth, supernatant, or water were added to the center. A 20 μL nematode suspension (about 100 J2s) was added to the center of the petri dish. The migration situation of nematodes was observed at 25°C. Nematodes that moved to sections 1-3 were considered repelled by the treatments, while those that moved to sections 4-6 were considered affinity (Cao et al., 2019). The behavior of the nematodes was determined by counting the numbers between the treated and control areas during the experimental period. At 12 h, the number of nematodes in two sections of each petri dish was counted. Each assay consisted of 10 plates and was repeated three times.

### 2.6 Invasion effect of Cq-C08 on *M. incognita* J2s

Cucumber seedlings in pots were treated with strain Cq-C08 fermentation broth (OD600 = 1.5), 50 mL per plant, with water as a mock. After 24 h, 300 J2s were inoculated into each pot. Each assay consisted of 8 plants and was repeated three times. After 48 h, the infected nematode numbers were observed using the acid fuchsin staining method (Bybd et al., 1983).

### 2.7 RT-qPCR analysis

Roots of hydroponic cucumber seedlings were immersed in 100 mL fermentation broth (OD600 = 0.4) of strain Cq-C08, with water as a control. Samples of cucumber root systems were collected at 0, 6, 12, 24, 48, and 72 h. Each time, the roots of 10 cucumber seedlings were treated, and each sample had three replicates. RNA samples of cucumber roots were extracted and reverse transcribed into cDNA using the Vazyme kit (Vazyme, Nanjing, China). Relative expression levels of cucumber immune regulatory genes involving SA, JA and ET signaling pathways were analyzed through RT-qPCR by using SYBR Green I (Vazyme, Nanjing, China) (Zhou et al., 2023), and determined using the 2^ΔΔCt^ method (Livak and Schmittgen, 2001). Primers for candidate marker genes were shown in Supplementary Table 1.

### 2.8 Split-root test

Split-root test assays were following previous methods (Tian et al., 2022; Yin et al., 2021). Cucumber seedlings at the one-leaf stage were transplanted into a split-root device, which was assembled from two adjacent pots cultivated at 28°C. Inoculation treatment was carried out at the two-leaf stage. The pot on the right side was inoculated with 50 mL strain Cq-C08 fermentation broth, while water was used as a control. Samples of cucumber root systems of the left pot were collected at 0, 6, 12, 24, 48, and 72 h. Relative expression levels of cucumber immune regulatory genes involving SA, JA and ET signaling pathways were analyzed through RT-qPCR by using SYBR Green I. After 48 h, 300 J2s were inoculated on the left pot. Each treatment consisted of 15 pots and was repeated three times independently. Six weeks after nematode inoculation, plant symptoms were observed, and galls and egg masses were counted.

### 2.9 Transcriptome analysis

Cucumber seeds were surface sterilized with 0.5% NaOCl and planted in pots filled with culture medium (peat/vermiculite, 2:1). At the two-leaf stage, 50 mL strain Cq-C08 fermentation broth (OD600 = 0.5) were inoculated into each pot, while water was used as a mock control. After 24 h, cucumber roots were inoculated with J2s. At 48 h post-inoculation with Cq-C08, root samples were taken for transcriptome sequence analysis.

Seq was conducted using the Illumina sequencing platform at Shanghai Personalbio Technology Co., Ltd. (Shanghai, China). The sequence data were aligned to the cucumber reference genome using HISAT2, an upgraded version of TopHat2.^1^ The clean reads were deposited in the NCBI Sequence Read Archive under accession number PRJNA1193783.Subsequent analyses were conducted using Genescloud.^2^ Based on the alignment results, the expression level of each gene was calculated, and the differential analysis was conducted using Deseq2. The conditions for the differentially expressed genes (DEGs) were selected as: expression difference multiple | log2FoldChange | > 1, significant *P*-value < 0.05. The number of up-regulated and down-regulated DEGs was counted for each comparison group. Gene ontology (GO) enrichment analysis was performed using cluster Profiler. During the analysis, genes annotated with GO terms were used to calculate the list and number of genes for each term. Then, the hypergeometric distribution method was performed to calculate the *P*-value with a significant threshold of *P*-value < 0.05.

ClusterProfiler was used for KEGG enrichment analysis. DEGs annotated with KEGG pathways were employed to calculate the number of genes associated with each pathway. The *P*-value for each pathway was determined using the hypergeometric distribution method, with a significance threshold of *P*-value < 0.05.

## 3 Results

### 3.1 Strain Cq-C08 was identified as *Candida quercitrusa*

**FIGURE 1:**
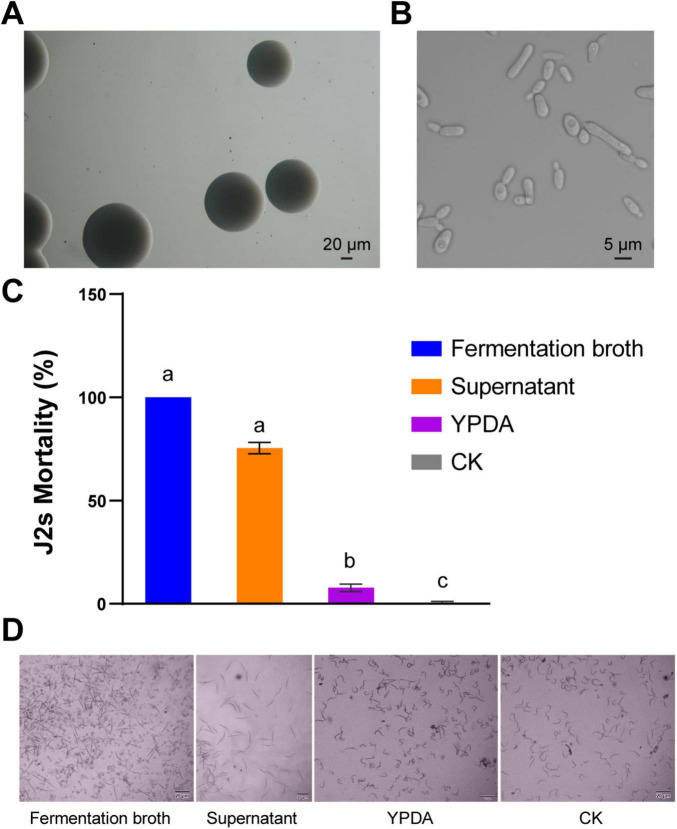
Morphological characteristics of the *Candida quercitrusa* strain Cq-C08 and its nematicial activity *in vitro*. **(A)** Colony characteristics of the strain Cq-C08. **(B)** Cell characteristics of the strain Cq-C08. Scale bars=20 μm **(A)** and 5 μm **(B)**. **(C)** Nematicial activity results of Cq-C08 *in vitro*. Mortality rates were determined by exposing 300 J2s of *Meloidogyne incognita* to the strain Cq-C08 fermentation broth, supernatant, YPDA and water (CK) in 24-well plates at 28°C for 12 h. Date with different letters in column chart indicated significant difference (One-way ANOVA followed by Tukey’s multiple range test at *P*< 0.01). **(D)** Phenotypes of *M. incognita* J2s after treatment with Cq-C08 fermentation broth, supernatant, YPDA and CK. Scale bars=20 μm.

Strain Cq-C08 was identified by morphological and molecular biological methods. On YPDA medium, strain Cq-C08 colonies were creamy white with a smooth surface and neat edges (Figure 1A). Under a microscope, cells were round or oval, with a size of 3 μm × (5-30 μm), budding and reproducing, and capable of forming pseudohyphae (Figure 1B). NL1F and NL4R primers were used to amplify partial 26S ribosomal DNA of strain Cq-C08, obtaining a sequence with 609 base pairs (Supplementary Figure 1A). BLAST analysis of the 26S rDNA sequence from strain Cq-C08 against NCBI GenBank revealed a 99% similarity to *Candida quercitrusa* (GenBank: AM160627.1 (Supplementary Figure 1B). Taken together, the strain was identified as *C. quercitrusa*, which has been deposited into the China General Microbiological Culture Collection Center (Beijing, China, with accession number CGMCC No. 2589.

### 3.2 Strain Cq-C08 exhibits high nematicidal activity *in vitro*

The cell culture plates were used to evaluate the nematicidal activity of strain Cq-C08 culture solution. The fermentation broth and supernatant of Cq-C08 exhibited high-level nematicidal activity against *Meloidogyne incognita* J2s, with mortality rates of 100 and 76% at 12 h, respectively. In contrast, after 12 h, the nematodes exhibited different morphologies, with almost no mortality observed in the water control group (Figures 1C,D). These results demonstrate that strain Cq-C08 has high-level nematicidal activity against *M. incognita*.

### 3.3 Strain Cq-C08 has significant control effects on *M. incognita*

**FIGURE 2:**
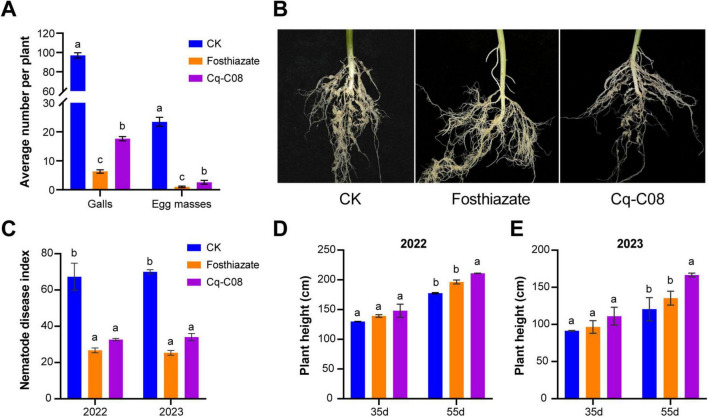
Control effect of the strain Cq-C08 on *M. incognita* in the pot and plot experiments. **(A)** The strain Cq-C08 showed significant control activity to *M. incognita* in the pot experiments. The average number of galls and egg masses per root system (with different treatment) was counted after inoculation with the *M. incognita* for 35 days. **(B)** Phenotypes of cucumber root symptoms under different treatments. **(C)** Nematode disease index in plot experiments of 2022 and 2023. **(D,E)** The cucumber height statistics results at 35 and 55 days post treatments for the year 2022 and 2023. Date with different letters in column chart indicated significant difference (One-way ANOVA followed by Tukey’s multiple range test at *P*< 0.05).

Potted experiments with nematode inoculation were used to determine the control effect of strain Cq-C08 on root-knot nematodes disease. In the water treatment (CK), the cucumber roots had an average of 97.0 ± 2.3 galls and 23.5 ± 1.3 egg masses per plant. However, data analysis showed that fosthiazate and Cq-C08 fermentation broth treatments significantly reduced the galls number, with an average of 6.3 ± 0.5 and 17.7 ± 0.6 per plant, respectively. Simultaneously, the average number of egg masses was 1.0 ± 0.2 and 2.6 ± 0.5 (Figures 2a,b). Thus, compared to the water control, the strain Cq-C08 control effect against *M. incognita* was 81.7%, while fosthiazate had a higher control effect of 93.5%. These results demonstrated that strain Cq-C08 had potential and value in controlling *M. incognita* disease.

Field plot assays were further conducted to validate the control effects of strain Cq-C08 against *M. incognita*. Two plot experiments were conducted in September 2022 and June 2023. In 2022 and 2023, the nematode disease index was 32.7 ± 0.9 and 34 ± 2.8. After treatment with strain Cq-C08 and fosthiazate, the disease indexes were 26.7 ± 1.8 and 25.3 ± 1.9, respectively (Figure 2C and Supplementary Figure 2). Compared to the water control, the strain Cq-C08 disease index was reduced by 51.5 and 51.4%, while fosthiazate showed a reduction of 63.4 and 63.8%. Interestingly, in the Cq-C08 treatment, plant height increased by 10.6% ± 3.3 and 38.2% ± 2.5 in 2022 (Figure 2D) and 2023 (Figure 2E), respectively. These results demonstrate that strain Cq-C08 shows a stable control effect on root-knot nematodes and could promote plant growth.

### 3.4 Strain Cq-C08 showed significantly repellence against *M. incognita*

**FIGURE 3:**
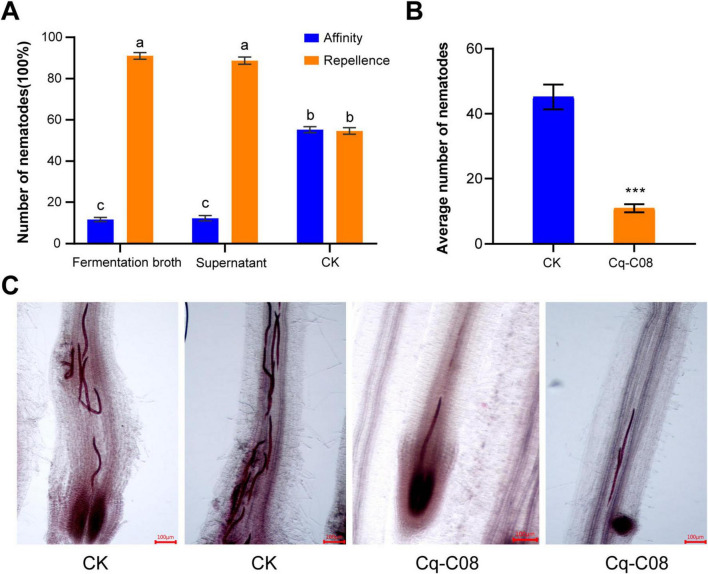
The strain Cq-C08 affected *M. incognita* activity. **(A)** Repellence analysis of strain Cq-C08 against the J2s of *M. incognita*. The test was carried out in square petri dishes with water agar and inoculated with 100 J2s of *M. incognita*. After 12 h, the number of nematodes at different positions were counted. Date with different letters in column chart indicated significant difference (One-way ANOVA followed by Tukey’s multiple range test at *P*< 0.001). **(B)** Quantification of the number of J2s infected in cucumber roots after treatment with strain Cq-C08 and water. Asterisks indicate significant differences relative to the mean ±SD values for the control treatment in Student’s *t*-test (*P*< 0.001). **(C)** Images of cucumber roots treated with strain Cq-C08 and water after acid fuchsin staining. Scale bars=100 μm.

To clarify the functions of strain Cq-C08 on *M. incognita*, we tested the chemotaxis analysis of Cq-C08 fermentation broth to *M. incognita* J2s (Supplementary Figure 3). Compared with water treatment, the number of nematodes attracted by fermentation broth and supernatant reduced by 91 and 88.7% at 12 h, respectively (Figure 3A). The results indicate that *M. incognita* J2s are significantly repelled by the Cq-C08 fermentation broth and supernatant.

### 3.5 Strain Cq-C08 reduced the infection ability of *M. incognita* J2s

To further determine the repellent effect of Cq-C08 on nematodes, acid fuchsin staining was performed to detect changes in the number of early infections of *M. incognita* J2s in cucumber roots after treatment with strain Cq-C08. Results showed that in the control treatment the number of nematodes was 45.2 ± 5.4, while in the Cq-C08 treatment was 10.9 ± 1.7 (Figures 3B,C). The results indicate that strain Cq-C08 can restrict the invasion of *M. incognita* J2s.

### 3.6 Strain Cq-C08 induced plant resistance to *M. incognita*

**FIGURE 4:**
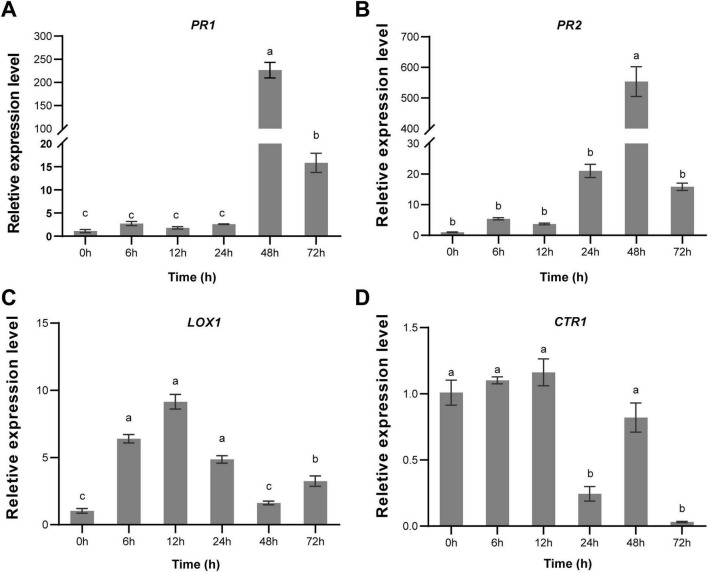
Expression profile of cucumber marker genes involved in immune response induced by the strain Cq-C08. **(A)** *PR1* (Pathogenesis-related 1). **(B)** *PR2* (β-1,3-glucanase gene). **(C)** *L0X1* (Lipoxygenase). **(D)** *CTRL* The *actin* was used as an endogenous gene to normalize the data, and fold-change values (mean ± SE) were calculated by the 2^–ΔΔCt^ method. Date with different letters in column chart indicated significant difference (One-way ANOVA followed by Tukey’s multiple range test at *P*< 0.05).

To clarify whether Cq-C08 induces plant defense against nematodes, the expression levels of marker genes involved in defense signal pathways of cucumber were analyzed by RT-qPCR, including the pathogenesis-related gene (*PR1*) and β-1,3-glucanase gene (*PR2*), which encode PR proteins associated with the SA signaling pathway. Lipoxygenase (*LOX1*) was the first enzyme in the synthesis of the JA pathway. The *CTR1* gene encoded a protein most similar to the Raf family serine/threonine protein kinases, and was a negative regulatory factor involved in the ET signaling pathway. The analysis showed that *PR1* and *PR2* expression levels were significantly activated after treatment with strain Cq-C08, reaching their highest expression levels at 48 h (Figures 4a,b). Meanwhile, the *LOX1* gene was up-regulated, with expression levels peaking at 12 h (Figure 4C). However, the expression level of *CTR1* showed no significant difference at 12 h, but with a down-regulating trend (Figure 4D). These results demonstrate that Cq-C08 could activate SA and JA-mediated immune defense.

**FIGURE 5:**
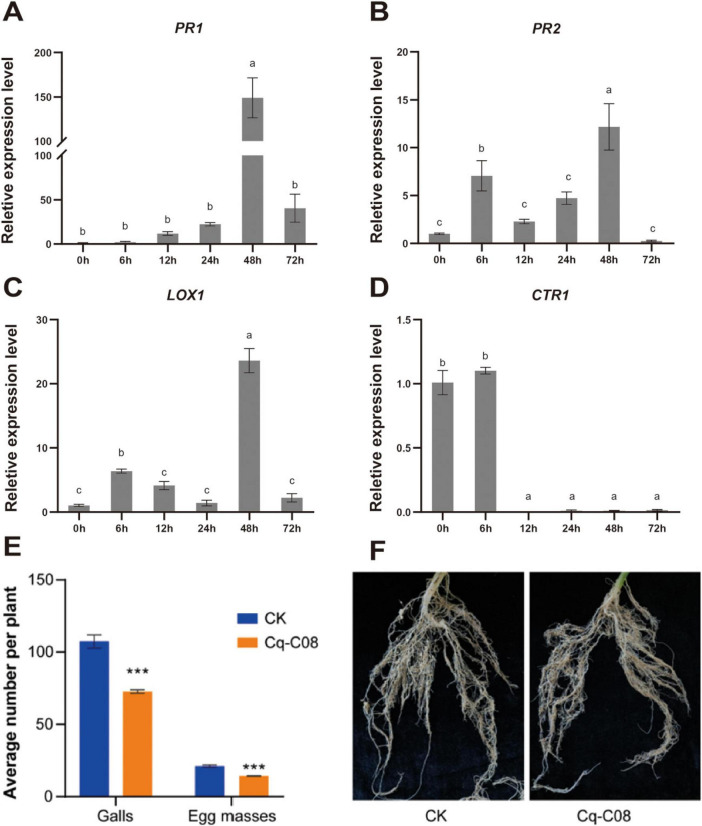
Control effect of the strain Cq-C08 on *M. incognita* in split-root test. **(A)** *PR1* (Pathogenesis-related 1). **(B)** *PR2* (β-1,3-glucanase gene). **(C)** *LOX1* (Lipoxygenase). **(D)** *CTR1.* **(E)** Quantification of the number of galls and egg masses per plant after treatment with water or Cq-C08. **(F)** Phenotype of cucumber roots after treatment with water or Cq-C08. The *actin* was used as an endogenous gene to normalize the data, and fold-change values (mean ± SE) were calculated by the 2^–ΔΔCt^ method. Asterisks indicate significant differences relative to the mean ±SD values for the control treatment in Student’s *t*-test (*P*< 0.001).

Split-root experiments were conducted to study whether strain Cq-C08 could induce cucumber resistance against *M. incognita* (Supplementary Figure 4). The RT-qPCR analysis showed that *PR1* and *PR2* expression levels were significantly activated after treatment with strain Cq-C08, reaching their highest expression levels at 48 h (Figures 5a,b). Meanwhile, the *LOX1* gene was up-regulated, with expression levels peaking at 48 h (Figure 5C). However, the expression level of *CTR1* showed no significant difference at 6 h, but with a down-regulating trend (Figure 5D). The split-root experiments results showed that cucumber roots treated with strain Cq-C08 had fewer galls and egg masses, with 72.7 ± 1.0 galls and 14.3 ± 0.2 egg masses per plant, while the control had 107.4 ± 3.8 galls and 21.1 ± 0.7 egg masses per plant (Figures 5E,B,F). Compared to the water control, the nematode disease index was reduced by 32.3% after strain Cq-C08 treatment. These results suggest that strain Cq-C08 can induce resistance in cucumber roots against *M. incognita*.

### 3.7 Strain Cq-C08 treatment promoted plant immune responses

**FIGURE 6:**
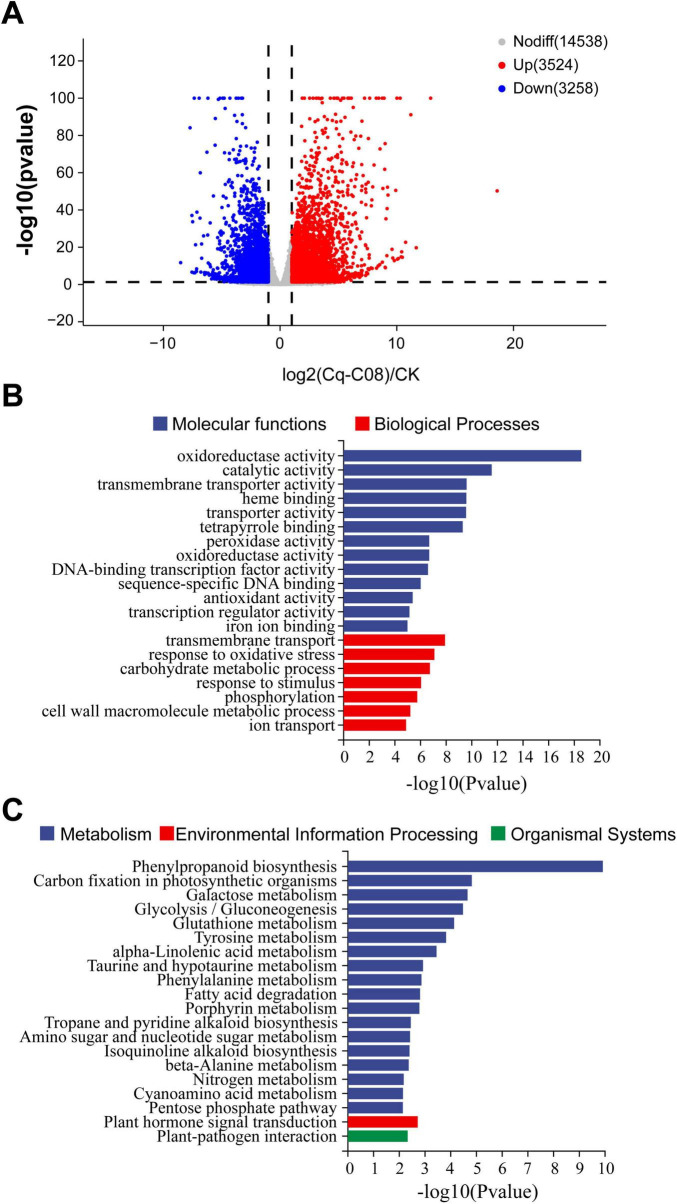
Analyzing the differentially expressed genes (DEGs) between Cq-C08 and CK treatments. **(A)** Volcano plot showed significantly changed DEGs. Each point in the graph represents a gene, with red indicating significantly up-regulated genes, blue indicating significantly down-regulated genes, and gray indicating genes with no significant difference. Points closer to the left, right, and top indicate more significant differences. The horizontal axis represents the fold change in gene/transcript expression between the two samples, and the vertical axis represents the *p*-value. **(B)** GO enrichment classification of DEGs treated with Cq-C08. Blue indicating Molecular functions (MF). Red indicating Biological Processes (BP). **(C)** KEGG enrichment classification of DEGs treated with Cq-C08. Blue indicating Metabolism. Red indicating Environmental Information Processing. Green indicating Organismal Systems.

To gain a comprehensive understanding of the changes in gene expression related to cucumber resistance after treatment with strain Cq-C08, the transcriptomes of cucumber roots inoculated with strain Cq-C08 and water were sequenced using the NovaSeq 6000 platform. A total of 6,782 differentially expressed genes (DEGs) were identified between CK and Cq-C08, with 3,524 up-regulated genes and 3,258 down-regulated genes (Figure 6A).

Gene Ontology (GO) molecular function enrichment was analyzed using DEGs identified by Cq-C08 treatment, including three aspects: biological processes (BP), molecular functions (MF), and cellular components (CC). The DEGs were mainly involved in BP such as oxidoreductase activity, catalytic activity, and transmembrane transporter activity. A smaller subset of DEGs was enrichment in CC, including transmembrane transport and oxidative stress response (Figure 6B).

The KEGG pathway enrichment analysis was performed to identify the primary biological functions carried out by DEGs, which involved 20 metabolic pathways, mainly including metabolism, environmental information processing, and organic systems. After treatment with Cq-C08, DEGs were largely enriched in metabolic pathways, including phenylpropanoid biosynthesis, carbon fixation in photosynthetic organisms, galactose metabolism, glycolysis, and glutathione metabolism. Additionally, there were environmental signal processing elements such as plant hormone signal transduction (Figure 6C).

**FIGURE 7:**
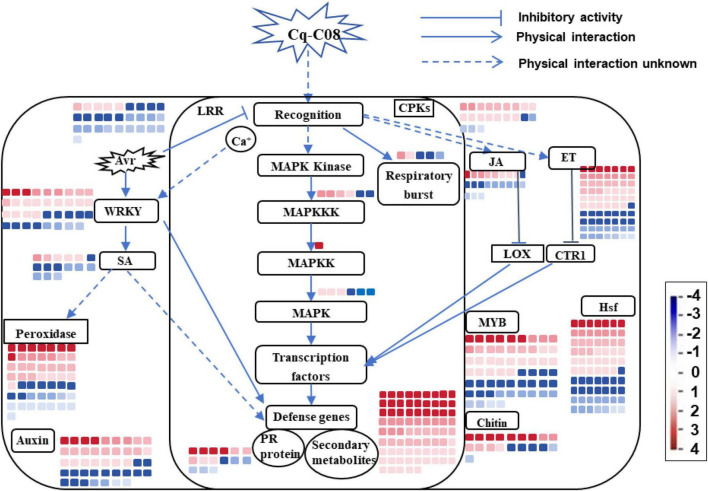
Mapman immune pathway analysis. The color bars represent the expression level and the number of DEGs. Red bars represent up-regulated genes, and blue bars represent down-regulated genes. The intensity of the color represents the fold change. The categorizations of functions for the DEGs are displayed within the boxed labels. The arrows depict the interactions that occur physically between various functional categories.

Using MapMan, the DEGs were clustered into various biological processes. Strain Cq-C08 induces plant resistance by modulating the SA, JA, and ET signaling pathways (Supplementary Table 2), as evidenced by the up-regulation of phenylalanine ammonia-lyase (*PAL*) in the SA pathway, lipoxygenase (*LOX*) in the JA pathway, and ethylene-responsive transcription factor (*ERF*) in the ET pathway. The mitogen-activated protein kinase (MAPK) pathway is also activated, with increases in *MAPKKK*, *MAPKK*, and *MAPK* genes (Supplementary Table 3). Receptor kinases are differentially regulated, with cysteine-rich receptor-like kinases up-regulated and LRR receptor-like kinases down-regulated. The immune response is further enhanced by the up-regulation of *WRKY*, *NAC*, and *MYB* transcription factors, and the activation of defense genes such as pathogenesis-related genes (*PR*), peroxidase (*POD*), and respiratory burst oxidase (*RBO*) (Supplementary Table 4). Strain Cq-C08 may also promote plant growth by up-regulating auxin production-related genes and induces the production of secondary metabolites with nematode-repellent properties, as eight Indole-3-acetic acid-amido synthetase genes (Supplementary Table 5) and genes related to small molecular secondary metabolites (terpenoids, flavonoids, sterols, organic acids, phenolics) are significantly up-regulated (Supplementary Table 6). Taken together, the activation of defense-related genes, development-related genes and secondary metabolites, along with the regulation of transcription factors after treatment with strain Cq-C08, suggests comprehensive positive responses of plants to nematode infection (Figure 7).

## 4 Discussion

Yeast strains, as biocontrol agents, possess numerous advantages. These include non-pathogenicity, broad-spectrum activity, competition with pathogens, production of antimicrobial compounds, induction of plant resistance, tolerance to environmental stress, ease of handling, compatibility with other agrochemicals, enhancement of plant growth, ecological and economic benefits, regulatory acceptance, and diversity for customized agricultural solutions (Cordero-Bueso et al., 2017; de Oliveira et al., 2023; Iturritxa et al., 2023). Previously, research identified seven yeast strains effective against *Meloidogyne incognita* in Flame Seedless grapevines, enhancing both productivity and fruit quality. Recently, a study revealed that the yeast *Papiliotrema terrestris* strain PT22AV exhibited promising nematicidal properties against *M. incognita* and enhanced the growth of tomato plants (D’Addabbo et al., 2024). This demonstrating the promising potential of yeasts as biological controls (Hashem et al., 2008). In this study, we isolated a *Candida quercitrusa* strain Cq-C08 from rhizosphere soil. This strain displayed potent nematicidal activity. Both pot and plot experiments indicated that strain Cq-C08 could effectively control M. incognita. These results suggest that strain Cq-C08 holds potential as a bio-nematicide against root-knot nematodes (RKNs).

Similarly, numerous biocontrol bacteria play crucial roles in activating hormone-triggered plant immune responses and promoting plant growth. Recently, researchers have shown that *Bacillus subtilis*, *Trichoderma longibrachiatum* H9, and *Pochonia chlamydosporia* can promote plant growth and enhance the plant immune system by regulating plant hormone levels, upregulating defense-related genes and proteins, thereby effectively preventing and controlling pests and diseases (Zhang et al., 2024; Yuan et al., 2019; Gouveia et al., 2023). We previously demonstrated that the effector protein MiMIF of *M. incognita* plays roles in SA synthesis and manipulates the rhizosphere microbiome to establish a parasitism relationship with hosts (Liu et al., 2023; Zhao et al., 2020). In this study, we discovered that strain Cq-C08 induced resistance against *M. incognita* in cucumber roots and activated the SA, JA, and ET signaling pathways. The expression levels of defense response genes *PR1* and *PR2* were significantly elevated at 48 h, while the expression levels of *LOX1* and *CTR1* were significantly increased at 12 h. The *PR1* and *PR2* genes encode PR proteins associated with the SA signaling pathway, with the PR1 gene being a key gene in systemic acquired resistance (SAR). *LOX1* ultimately leads to the biosynthesis of JA, and the activity of the *LOX* gene is triggered by nematode infection. In the split-root experiment, the number of galls on roots treated with strain Cq-C08 decreased by 32.3%. These results confirm that strain Cq-C08 activates SA and JA-mediated immune defense, promoting plant resistance to RKNs.

*In vivo* experiments revealed the potential of DH16 to control *M. incognita* populations and promote tomato plant growth, suggesting its multifunctional use as a nematicidal agent and a plant growth promoter (Kaur et al., 2016). In this study, we found that strain Cq-C08 could promote plant growth. Indole-3-acetic acid-amido synthetase was significantly up-regulated. This enzyme plays an important role in plants, promoting plant growth and development and being involved in defense responses mediated by SA and JA. Studies have shown that yeast has the potential as a biofertilizer and biopesticide, which can provide soluble nutrients to plants and promote plant growth by producing organic acids and indo-3-acetic acid (Hernández-Fernández et al., 2021).

Through comparative transcriptome analysis, it was also discovered that strain Cq-C08 could induce plant immune responses and produce secondary metabolites (SMs) to defend against *M. incognita*. Among these, phenylalanine ammonia-lyase (PAL) is a key enzyme in plant disease resistance, involved in the synthesis of lignin and SA (Zhu et al., 2024). The formation of lignin increases the thickness of the cell wall, creating a barrier to prevent pathogen invasion (He et al., 2020). In the comparative transcriptome analysis, terpenoids, ketones, alcohols, and other substances produced by small molecules such as terpene synthase, alcohol dehydrogenase (ADH), sterol, and SM exhibited repellent and toxic effects on RKNs. To resist pathogen attack, plants defend themselves by releasing toxins and penetrating the barrier. The synthesis genes of cucurbit-specific metabolic products, cucumisin and cucumber peeling cupredoxin, were both up-regulated. Interestingly, these compounds contribute to cucurbit disease resistance and stress regulation, enhancing plant resistance to pests and diseases by regulating the composition of the rhizosphere microbial community (Zhong et al., 2022). This may explain why strain Cq-C08 had a significant repellent effect on *M. incognita* and was able to reduce the nematode’s ability to invade plants. Therefore, strain Cq-C08 can prevent RKNs from invading plants.

In the defense signaling pathways, strain Cq-C08 can act as an elicitor, interact with receptor kinases, and participate in the plant’s defense response against pathogens through the MAPK cascade pathway. Finally, it activates defense genes via transcription factors. This includes defense genes related to basic immunity such as chitin, xyloglucan, and pectin, which are present in the plant cell walls and play key roles in the defense responses against pathogens and pests (Zhang et al., 2023). In this study, we demonstrated that cucumber basal immunity was enhanced after treatment with strain Cq-C08, contributing to the defense against nematode infection. A large number of stress response genes were up-regulated, such as heat stress transcription factors (Hsfs). Hsfs are a class of proteins that are rapidly expressed in organisms under external stress, such as high temperatures and chemical substances, and play a primary role in plant growth and stress resistance (Jacob et al., 2017). We speculate that strain Cq-C08 rapidly activates plant immune signaling pathways, integrating plant hormones and secondary metabolites, and promoting plant resistance to RKNs.

The split-root experiment revealed that *C. quercitrusa* Cq-C08 triggers *systemic resistance* in cucumber, as evidenced by elevated defense gene expression (*PR1*, *PR2*, *LOX1*) and reduced gall formation (32.3%) in untreated root tissues. While local (hydroponic) and systemic responses both peaked at 48 h, systemic gene activation likely involves delayed signal translocation. Mobile defense signals, such as SA or JA derivatives, may require time to traverse the plant vasculature, explaining the synchronized peak in systemic tissues. This contrasts with direct local responses, where rapid pathogen-associated molecular pattern (PAMP) recognition initiates immediate defense transcription. Systemic resistance offers practical advantages: a single application to one root zone could protect the entire plant, reducing labor and resource inputs. Notably, the activation of SA and JA pathways in systemic tissues suggests cross-protection against other pathogens, a trait observed in *Bacillus*-induced systemic resistance (Choudhary and Johri, 2009; Samaniego-Gámez et al., 2023). Unlike localized biocontrol agents (e.g., *Pseudomonas* spp.), Cq-C08’s systemic effect enhances its utility in field settings, where nematode distribution is heterogeneous. Furthermore, the durability of systemic resistance—evidenced by persistent defense gene upregulation—may reduce nematode adaptation pressures, a common issue with chemical nematicides. These findings position Cq-C08 as a multifaceted biocontrol agent, combining direct nematicidal activity with long-lasting plant immunity. Our study paves the way for further exploration into the practical application of strain Cq-C08 as a biocontrol agent, offering a promising avenue for sustainable agricultural practices.

## Data Availability

The datasets presented in this study can be found in online repositories. The names of the repository/repositories and accession number(s) can be found in the article/Supplementary material.

## Footnotes

1
http://ccb.jhu.edu/software/hisat2/index.shtml

2
https://www.genescloud.cn/home
