# Supplementary figures and images for: The Candida quercitrusa strain Cq-C08 induces plant resistance to root-knot nematodes
Source: Front Microbiol. 2025 Apr 17;16:1546583. doi: 10.3389/fmicb.2025.1546583 (PMC12043707; doi:10.3389/fmicb.2025.1546583)

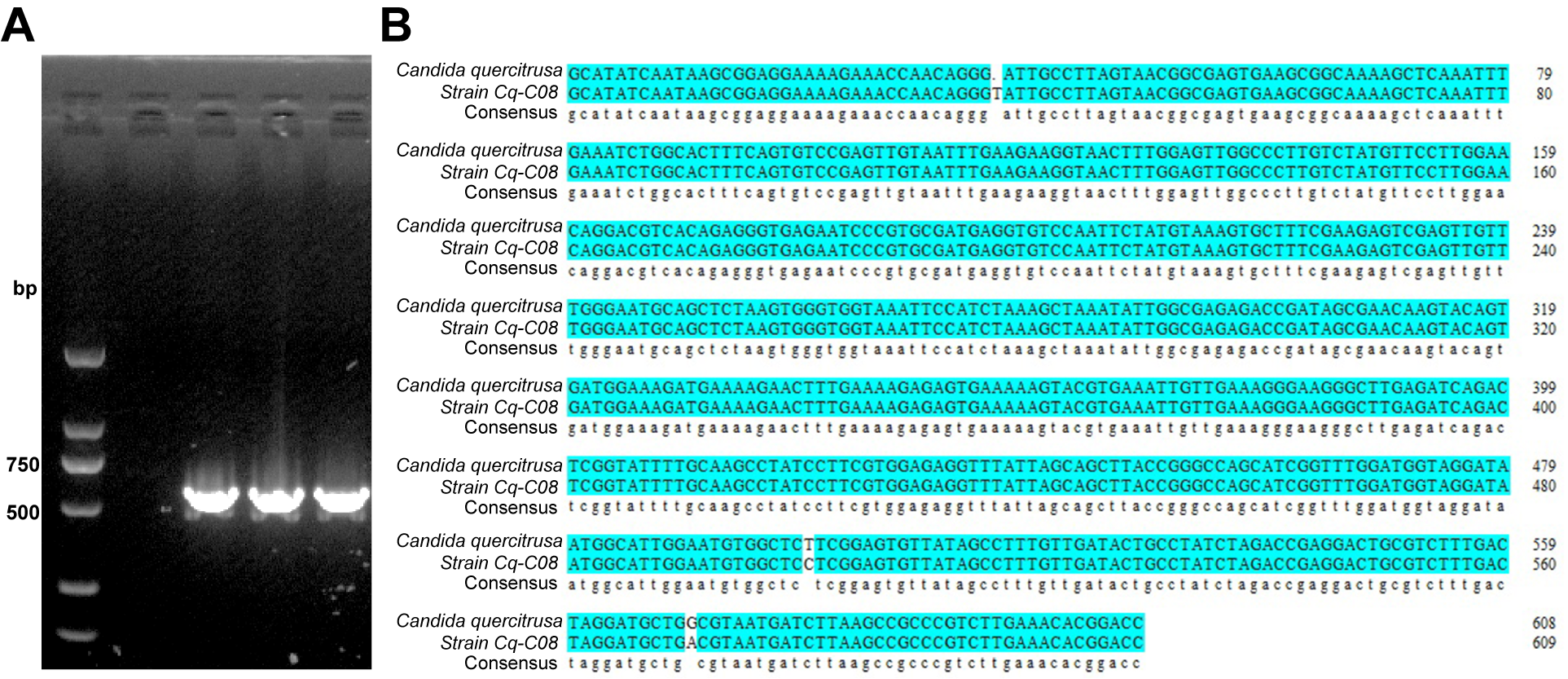

Supplement: Supplementary file 2 [file Image_1.tif]

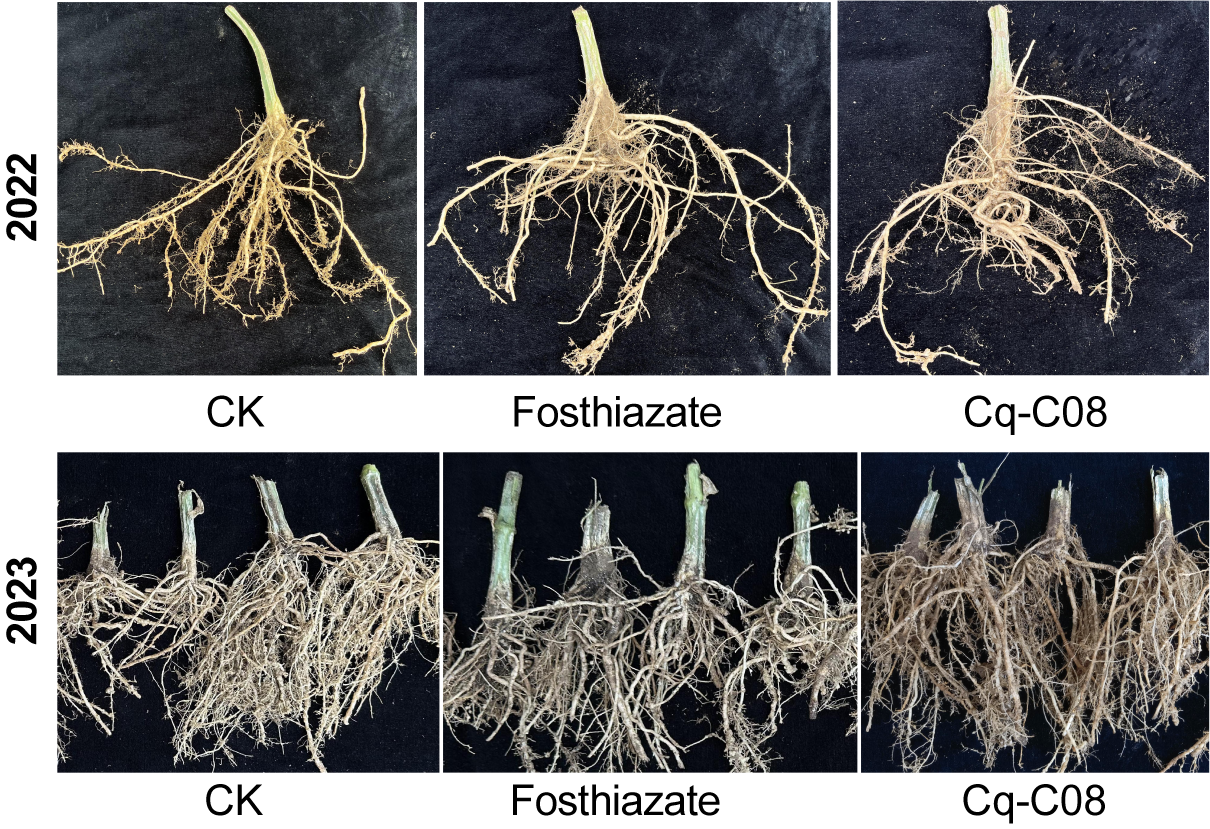

Supplement: Supplementary file 3 [file Image_2.tif]

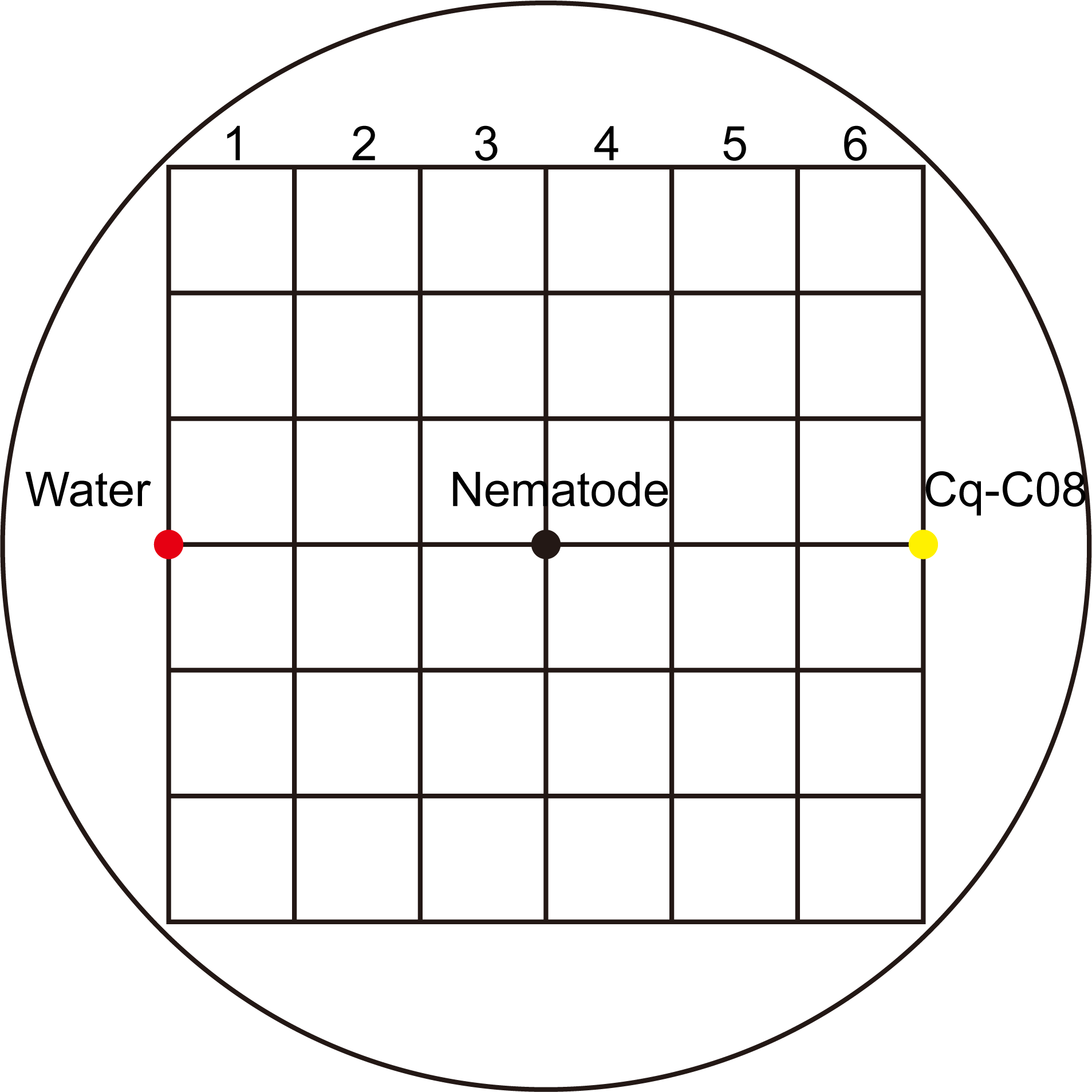

Supplement: Supplementary file 4 [file Image_3.tif]

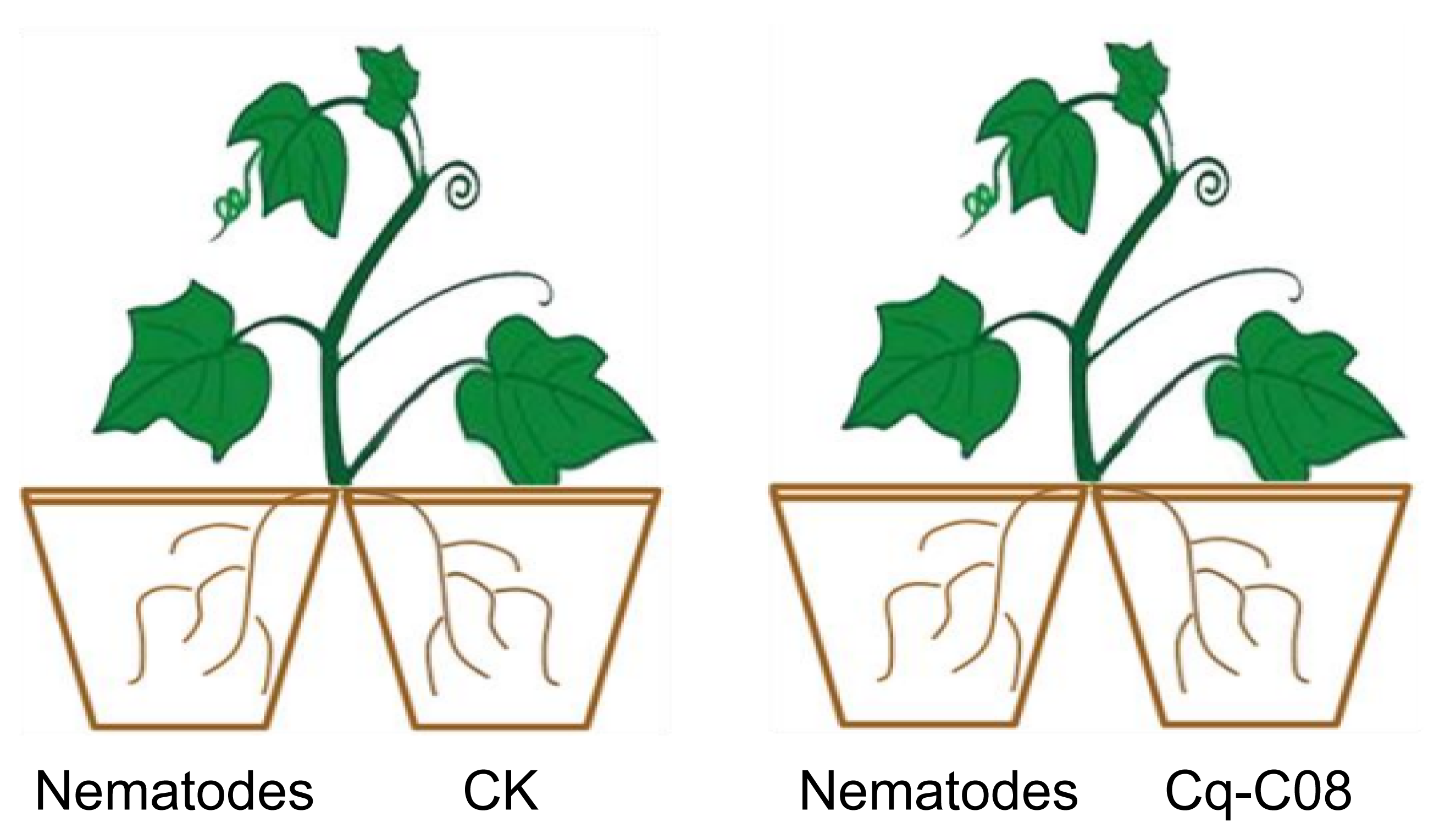

Supplement: Supplementary file 5 [file Image_4.tif]
